# Activation of the 5-hydroxytryptamine 4 receptor ameliorates tight junction barrier dysfunction in the colon of type 1 diabetic mice: Role of 5-HT _4_R in the tight junction barrier of T1D

**DOI:** 10.3724/abbs.2023137

**Published:** 2023-09-28

**Authors:** Shasha Hu, Yueting Kou, Xiaochen Liu, Weifang Rong, Hongxiu Han, Guohua Zhang

**Affiliations:** 1 Department of Anatomy and Physiology Shanghai Jiao Tong University School of Medicine Shanghai 200025 China; 2 Department of Pathology Tongji Hospital Tongji University Shanghai 200065 China

**Keywords:** 5-HT _4_R, diabetes, tight junction barrier, MLCK, ROCK1

## Abstract

Hyperglycemia drives dysfunction of the intestinal barrier. 5-Hydroxytryptaine 4 receptor (5-HT _4_R) agonists have been considered therapeutics for constipation in clnic. However, the roles of 5-HT _4_R activation in mucosa should be fully realized. Here, we investigate the effects of 5-HT _4_R activation on diabetes-induced disruption of the tight junction (TJ) barrier in the colon. Not surprisingly, the TJ barrier in diabetic mice with or without 5-HT _4_R is tremendously destroyed, as indicated by increased serum fluorescein isothiocyanate (FITC)-dextran and decreased transepithelial electrical resistance (TER). Simultaneously, decreased expressions of TJ proteins are shown in both wild-type (WT) and 5-HT _4_R knockout (KO) mice with diabetes. Notably, chronic treatment with intraperitoneal injection of a 5-HT _4_R agonist in WT mice with diabetes repairs the TJ barrier and promotes TJ protein expressions, including occludin, claudin-1 and ZO-1, in the colon, whereas a 5-HT _4_R agonist does not improve TJ barrier function or TJ protein expressions in 5-HT _4_R KO mice with diabetes. Furthermore, stimulation of 5-HT _4_R inhibits diabetes-induced upregulation of myosin light chain kinase (MLCK), Rho-associated coiled coil protein kinase 1 (ROCK1), and phosphorylated myosin light chain (p-MLC), which are key molecules that regulate TJ integrity, in the colonic mucosa of WT mice. However, such action induced by a 5-HT _4_R agonist is not observed in 5-HT _4_R KO mice with diabetes. These findings indicate that 5-HT _4_R activation may restore TJ integrity by inhibiting the expressions of MLCK, ROCK1 and p-MLC, improving epithelial barrier function in diabetes.

## Introduction

Hyperglycemia, a characteristic of the metabolic disease known as diabetes, is linked to a variety of dysfunctions. As an autoimmune T-cell-mediated condition, type 1 diabetes (T1D) is associated with disorders of the immune system, microbiota, and mucosal barrier in the gut [1]. Emerging evidence indicates that T1D-induced hyperglycemia results in the disruption of the intestinal barrier and risk of enteric infection through reprogramming epithelial cells [2].

The intestinal mucosa acts as a barrier between the host and luminal contents. The physical epithelial barrier can effectively prevent pathogens, toxins, antigens, and other harmful molecules from entering the lumen [3]. Increased intestinal permeability is a sign of epithelial dysfunction, which is linked to the disruption of tight junctions (TJs), the primary factor determining paracellular permeability [ 4, 5]. The TJ consists of multiple protein complexes, including claudins and occludin, transmembrane proteins, and zona occludens (ZOs), linking transmembrane and cytoplasmic proteins [6]. Reduced expression of ZO-1 in *db*/ *db* mice associated with intestinal dysfunction has been reported [2].

Peripheral 5-hydroxytryptamine (5-HT) produced by enterochromaffin cells plays an important role in motor, secretory and sensory functions in the gut by activating several 5-HT receptors [7]. Recently, close attention has been paid to the importance of the mucosal 5-HT _4_ receptor (5-HT _4_R). Stimulation of mucosal 5-HT _4_R evokes 5-HT release, goblet cell degranulation and Cl ^‒^ secretion, and activation of mucosal 5-HT _4_R can produce antinociceptive action [8]. The activation of epithelial 5-HT _4_R can reduce inflammation by promoting epithelial cell proliferation and wound healing [9]. 5-HT _4_R agonist protects the colon against diabetes-induced bacterial translocation by triggering mucin 2 production [10]. Therefore, it is reasonable to speculate that activation of 5-HT _4_R may restore disruption of TJ barrier in diabetes.

It is generally accepted that phosphorylation of myosin light chain (p-MLC) induces contraction of the perijunctional actomyosin ring, which leads to the internalization of TJ proteins and disruption of TJ barrier. MLC kinase (MLCK) and Rho-associated coiled coil protein kinase (ROCK) can phosphorylate MLC directly [11]. Melatonin has been shown to ameliorate intestinal epithelial permeability in diabetic rats by inhibiting either MLCK or ROCK signaling [12]. Furthermore, PKA has been linked to the downregulation of MLC phosphorylation through the inhibition of MLCK and ROCK signaling in endothelial barrier function [ 13, 14]. 5-HT _4_R is a G protein-coupled receptor which activates the intracellular cAMP-PKA substrate when stimulated.

In the present study, we investigated whether 5-HT _4_R activation ameliorates diabetes-driven TJ barrier dysfunction by inhibiting MLC phosphorylation mediated by either MLCK or ROCK1.

## Materials and Methods

### Animals

Adult (~10 week) male 5-HT _4_R knockout (KO) and age-matched wild-type (WT) C57BL/6 mice were used in the current study. The 5-HT _4_R KO mice (GeneBank Accession Number: NM_008313.4; Ensembl: ENSMUSG00000026322) were purchased from Cyagen Biosciences Inc. (Guangzhou, China) and bred at the Animal Facility of Basic Medical Sciences, Shanghai Jiao Tong University. The success of 5-HT _4_R KO in mice is shown in Supplementary Figure S1. The animals were housed in a temperature-controlled room (25°C) with specific pathogen-free conditions and *ad libitum* access to water and food. All experimental protocols were approved by the Ethics Committee of Shanghai Jiao Tong University School of Medicine (A-2022-051).

### Induction of diabetes and experimental design

Mice were injected intraperitoneally with streptozotocin (STZ; 150 mg/kg; Sigma, St Louis, USA) dissolved in 0.01 M cold citrate buffer (pH 4.0) to establish type 1 diabetes. Nondiabetic mice were given the same vehicle volume. Blood glucose was tested weekly using the glucose-oxidase test strip and a reflectance meter (Roche Diagnostics GmbH, Clarecastle, Ireland). Animals showing sustained hyperglycemia were used for the subsequent experiments. After 2 weeks of STZ injection, a highly selective 5-HT _4_R agonist RS67333 (1 mg/kg; Tocris, Ellisville, USA) was intraperitoneally administered once every other day six times based on our preliminary experiment and a previous study [15]. The following experiments were performed 24 h after the last injection.

### Measurement of serum FITC-dextran

On the test day, the mice were fasted for 4 h, and then 4 kDa fluorescein isothiocyanate (FITC)-dextran (440 mg/kg) was administered by gavage. Blood was collected from the heart 3 h after gavage, centrifuged at 10,000 *g* for 10 min and left to stand overnight at 4°C. The fluorescence of serum FITC-dextran was measured using a fluorescence plate reader (Gene Company Limited, Shanghai, China) with 485 nm excitation and 535 nm emission.

### Ussing chamber experiment

Transepithelial electrical resistance (TER) was measured with a P 2300 Ussing chamber system (Warner Instruments, Holliston, USA) according to the manufacturer’s instructions. In brief, Eusebio chambers were calibrated, and the flat sheet of distal colon excised from mice was immediately mounted onto the chamber pin. Voltage clamp recordings were performed. Tissues were maintained at 37°C in oxygenated (95% O _2_ with 5% CO _2_) Krebs solution (120 mM NaCl, 5.9mM KCl, 1.2mM NaH _2_PO4 _2_, 1.2 mM MgSO _4_, 15.4 mM NaHCO _3_, 2.5 mM CaCl _2_, and 11.5 mM glucose) throughout the recording period [16].

### Immunofluorescence (IF) microscopy

Mice were transcardially perfused with 0.1 M phosphate buffer (PB) followed by 4% paraformaldehyde (PFA) under deep anesthesia. The distal colons were removed, postfixed in 4% PFA and embedded with OCT. The sections (10 μm) were blocked with 10% normal goat serum in 0.05 M PBS and were incubated with one of the following primary antibodies at 4°C overnight: 1) rabbit anti-occludin (1:1500; GB111401; Servicebio, Shanghai, China); 2) rabbit anti-claudin-1 (1:500; GB11032; Servicebio); and 3) rabbit anti-ZO-1 (1:1500; GB111402; Servicebio). The following day, sections were incubated with goat anti-rabbit Alexa Fluor 488 secondary antibody (1:1000; Molecular Probes-Invitrogen, Eugene, USA) at room temperature for 1 h. The sections were viewed under a fluorescence microscope (Leica DM2500; Leica, Wetzlar, Germany), and digital images were captured using Leica Application Suite version 4.3 (Leica). Integrated density was measured to evaluate fluorescent signals using ImageJ ( http://rsb.info.nih.gov/ij/).

### Western blot analysis

The distal colon was longitudinally opened, and the mucosa was removed by cell scraping. The mucosa was homogenized with lysis buffer (20 mM Tris-HCl, pH 8.0, containing 150 mM NaCl, 1 mM EDTA, 1 mM PMSF), and protease inhibitor cocktail and phosphatase inhibitor. Equal amounts of protein were separated on a 10% Tris-glycine gel and then transferred to a PVDF membrane (Merck Millipore, Darmstadt, Germany). Blots were incubated with one of the following antibodies: 1) rabbit anti-occludin (1:2000; #91131; Cell Signaling, Beverly, USA); 2) rabbit anti-claudin-1 (1:2000; T56872; Abmarts, Berkeley Heights, USA); 3) rabbit anti-MLCK (1:500; GB113358; Servicebio); 4) rabbit anti-ROCK1 (1:500; GB111691; Servicebio); and 5) rabbit anti-pMLC (1:500; TA8618; Abmarts), followed by incubation with HRP-conjugated secondary antibody (1:2000; #1706515; Bio-Rad, Hercules, USA). The density of specific bands was measured with ImageJ and was normalized against a loading control (β-actin).

### Statistical analysis

Data are presented as the mean±standard error and then statistically analyzed using GraphPad Prism version 9.0 (GraphPad, La Jolla, USA). The percentage change in either serum FITC-dextran or immunofluorescent signal was quantified when the mean value in the nondiabetic group was considered 100. One-way or two-way ANOVA with post hoc Tukey’s multiple comparisons test was performed as appropriate. *P*<0.05 was considered statistically significant.

## Results

### 5-HT _4_R activation attenuates diabetes-induced dysfunction in the TJ barrier

**Figure 1:**
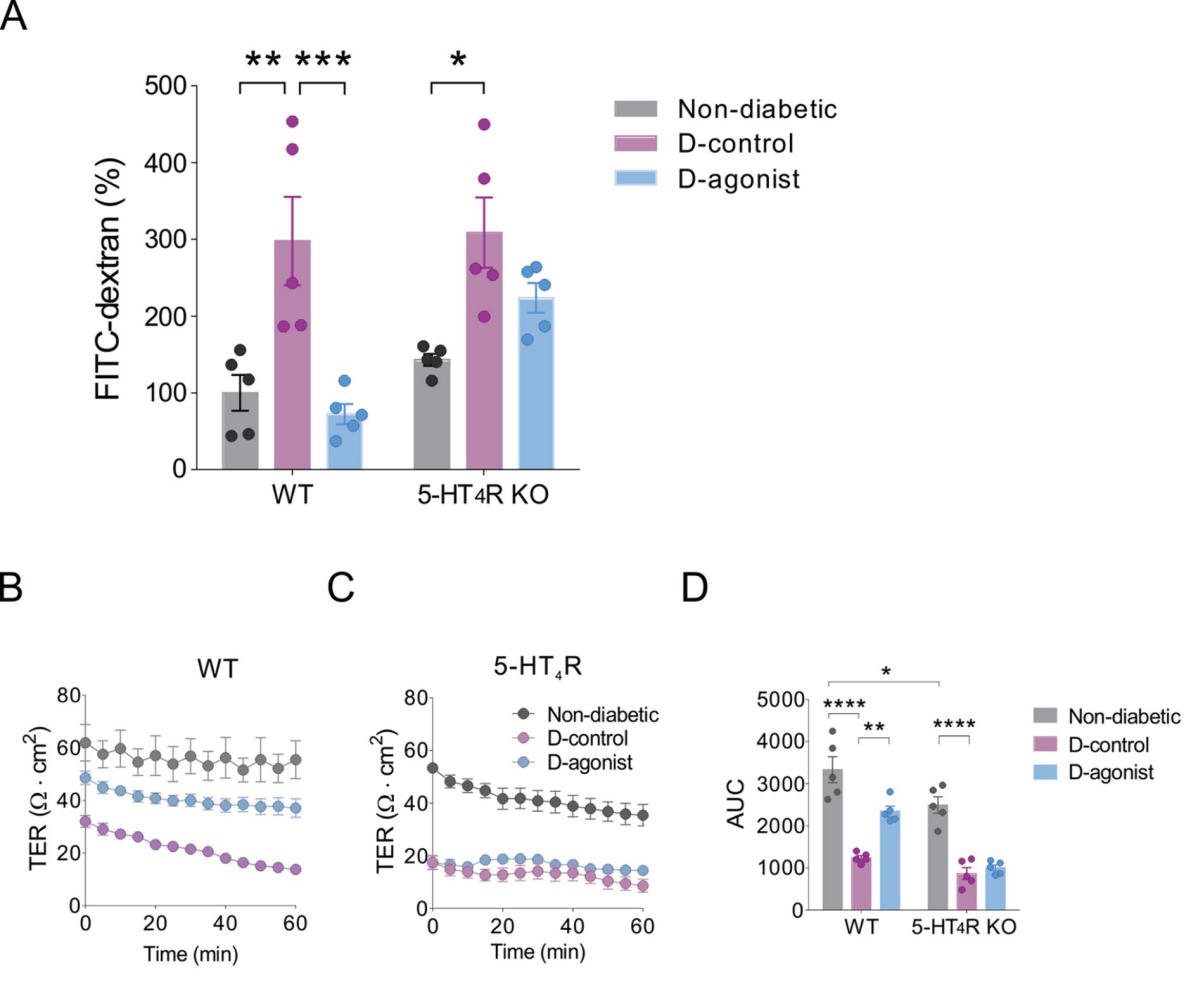
Figure 1 Activation of 5-HT _4_R alleviates diabetes-induced dysfunction of the tight junction barrier in mice (A) Effects of a 5-HT 4R agonist on serum FITC-dextran 4 weeks after STZ injection in WT and 5-HT4R KO mice. *P<0.05, **P<0.01, ***P<0.001, two-way ANOVA with Tukey’s multiple comparisons test (n=5 mice for each group). (B‒D) Effects of a 5-HT4R agonist on distal colon TER 4 weeks after STZ injection in WT and 5-HT4R KO mice. Ussing chamber recordings from WT and 5-HT4R mice with nondiabetic, diabetic-control and diabetic-5-HT4R agonist treatments are shown in (B,C). The area under the curve (AUC) for the statistical analysis of TER is shown in (D). *P<0.05, **P<0.01, ****P<0.0001, two-way ANOVA with Tukey’s multiple comparison test (n=5 mice for each group). D: diabetic.

To determine whether 5-HT _4_R activation affects TJ barrier function in diabetic mice, we detected serum FITC-dextran and TER levels in the distal colon upon chronic treatment with the 5-HT _4_R agonist RS67333 (1 mg/kg) beginning 2 weeks after STZ injection. The TJ barrier was destroyed, as indicated by higher serum FITC-dextran levels in both WT and 5-HT _4_R KO mice with diabetes than in non-diabetic mice. Treatment with chronic RS67333 significantly reduced the diabetes-induced increase in serum FITC-dextran in WT mice. To confirm that RS67333-mediated protection occurs via the 5-HT _4_R receptor, experiments were performed in mice with genetic deletion of 5-HT _4_R. As expected, the protective effect of RS67333 on the TJ barrier against diabetes was not observed in mice lacking 5-HT _4_R ( Figure 1A). In addition, TER decline in the distal colon, which is a direct index of a disrupted TJ barrier, was observed in both WT and 5-HT _4_R KO mice with diabetes. The 5-HT _4_R agonist alleviated diabetes-induced TER decline in the distal colon in WT mice, but this effect was absent in 5-HT _4_R KO mice, further providing evidence that 5-HT _4_R activation protects the TJ barrier against diabetes ( Figure 1B–D). However, RS67333 did not affect diabetes-induced changes in body weight and blood glucose in either WT or 5-HT _4_R KO mice ( Table 1).

**Table:** **Table 1** Effects of a 5-HT _4_R agonist on diabetes-induced alterations in body weight and blood glucose levels in WT and 5-HT _4_R KO mice

Group	Body weight (from day 0)		Blood glucose (mM)
	Day 0	Day 14	Day 28		Day 0	Day 14	Day 28
WT Nondiabetic	100	105.2±2.18	111.3±2.05		9.1±0.26	10.2±0.31	9.0±0.19
WT D-control	100	82.3±4.32 ^****^	82.4±1.99 ^****^		9.1±0.42	28.6±1.06 ^****^	32.9±0.45 ^****^
WT D-agonist	100	80.4±3.55	81.3±2.15		10.5±0.45	29.1±0.97	32.8±0.40
KO Nondiabetic	100	106.5±5.17	110.9±5.64		9.1±0.30	8.7±0.23	9.4±0.41
KO D-control	100	84.4±1.42 ^****^	83.1±2.94 ^****^		10.2±0.36	27.6±1.67 ^****^	31.5±1.06 ^****^
KO D-agonist	100	83.8±2.32	82.6±2.86		8.8±0.4	25.6±2.03	31.9±1.38

**** *P*<0.0001, vs nondiabetic.

### 5-HT _4_R activation inhibits diabetes-induced downregulation of TJ proteins in the colon

To investigate whether 5-HT _4_R-mediated protection from diabetes-driven TJ barrier disruption is involved in 5-HT _4_R-promoted expression of TJ proteins, we examined the effects of a 5-HT _4_R agonist on occludin, claudin-1, and ZO-1 expressions in the diabetic colon by IF staining and western blot analysis.

**Figure 2:**
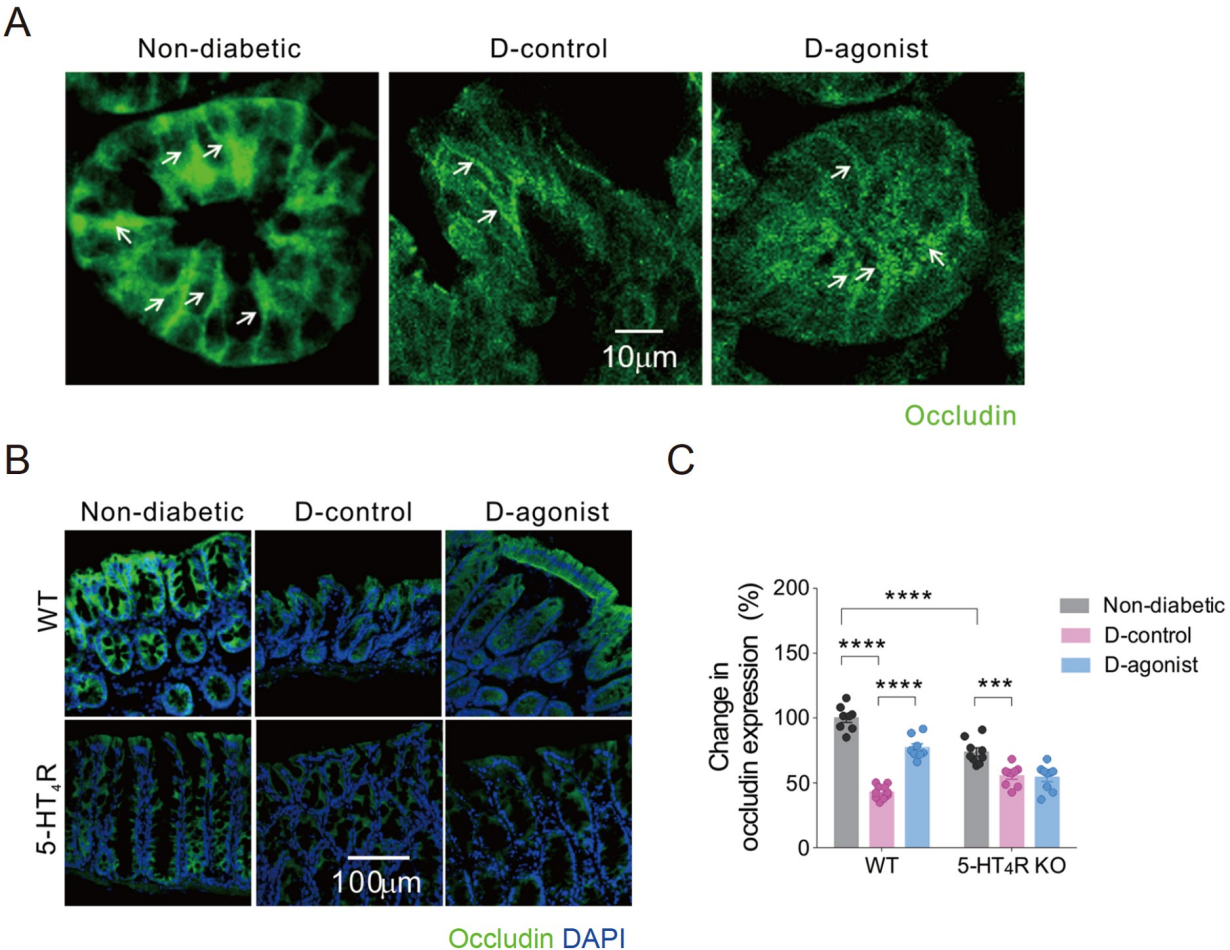
Figure 2 Activation of 5-HT _4_R interferes with diabetes-induced decrease in immunofluorescence intensity of occludin in the colon of mice (A) Representative photomicrographs of IF staining of occludin in colon epithelial cells from WT mice, indicated by white arrows. Scale bar: 10 μm for all three images. (B) Representative photomicrographs of IF staining of occludin (green) with DAPI (blue) in colon sections of WT and 5-HT4R KO mice. Scale bar: 100 μm for all six images. (C) Quantification of the IF intensity of occludin. *** P<0.001, ****P<0.0001, two-way ANOVA with Tukey’s multiple comparisons test (n=7–10 sections from 3–4 mice for each group). D: diabetic.

As shown in nondiabetic mice, occludin IF staining, characterized by a band-like pattern, was evenly localized on the lateral membrane of epithelial cells in the colon. On the other hand, weak staining and uneven occludin distribution were observed in mice with diabetes. Notably, the dense staining pattern of occludin was repaired by the 5-HT _4_R agonist in mice with diabetes ( Figure 2A). Expression of occludin in the colon was downregulated by diabetes in both WT and 5-HT _4_R KO mice. The 5-HT _4_R agonist restored occludin expression in diabetic WT mice, while it failed to exert such an effect on diabetic mice with 5-HT _4_R deletion. Even in nondiabetic mice, occludin expression was lower in 5-HT _4_R KO mice than in WT mice ( Figure 2B,C).

**Figure 3:**
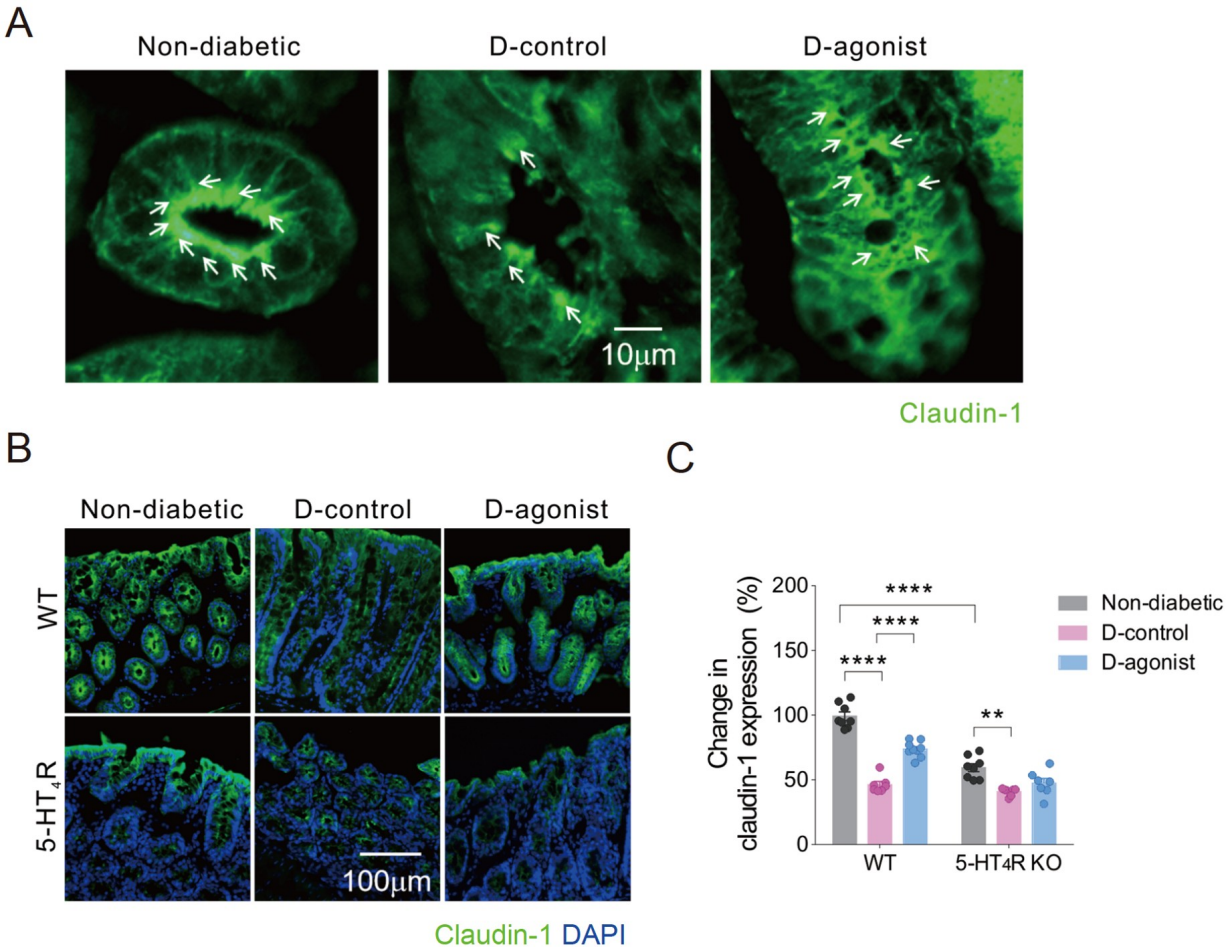
Figure 3 Activation of 5-HT _4_R restricts diabetes-induced decrease in immunofluorescence intensity of claudin-1 in the colon of mice (A) Representative photomicrographs of IF staining of claudin-1 in colon epithelial cells from WT mice, indicated by white arrows. Scale bar: 10 μm for all three images. (B) Representative photomicrographs of IF staining of claudin-1 (green) with DAPI (blue) in colon sections of WT and 5-HT4R KO mice. Scale bar: 100 μm for all six images. (C) Quantification of the IF intensity of claudin-1. ** P<0.01, ****P<0.0001, two-way ANOVA with Tukey’s multiple comparisons test (n=7–10 sections from 3–4 mice for each group). D: diabetic.

IF staining revealed that claudin-1 was continuously localized on the lateral membrane of epithelial cells, particularly concentrated in the apical part, in the colon of nondiabetic mice. However, the scattered distribution of claudin-1 was observed in colon epithelial cells in diabetic mice. Strikingly, continuous distribution partially reappeared in diabetic mice treated with the 5-HT _4_R agonist ( Figure 3A). The quantitative change in IF staining of claudin-1 was similar to that of occludin in both WT and 5-HT _4_R KO mice ( Figure 3 B,C).

**Figure 4:**
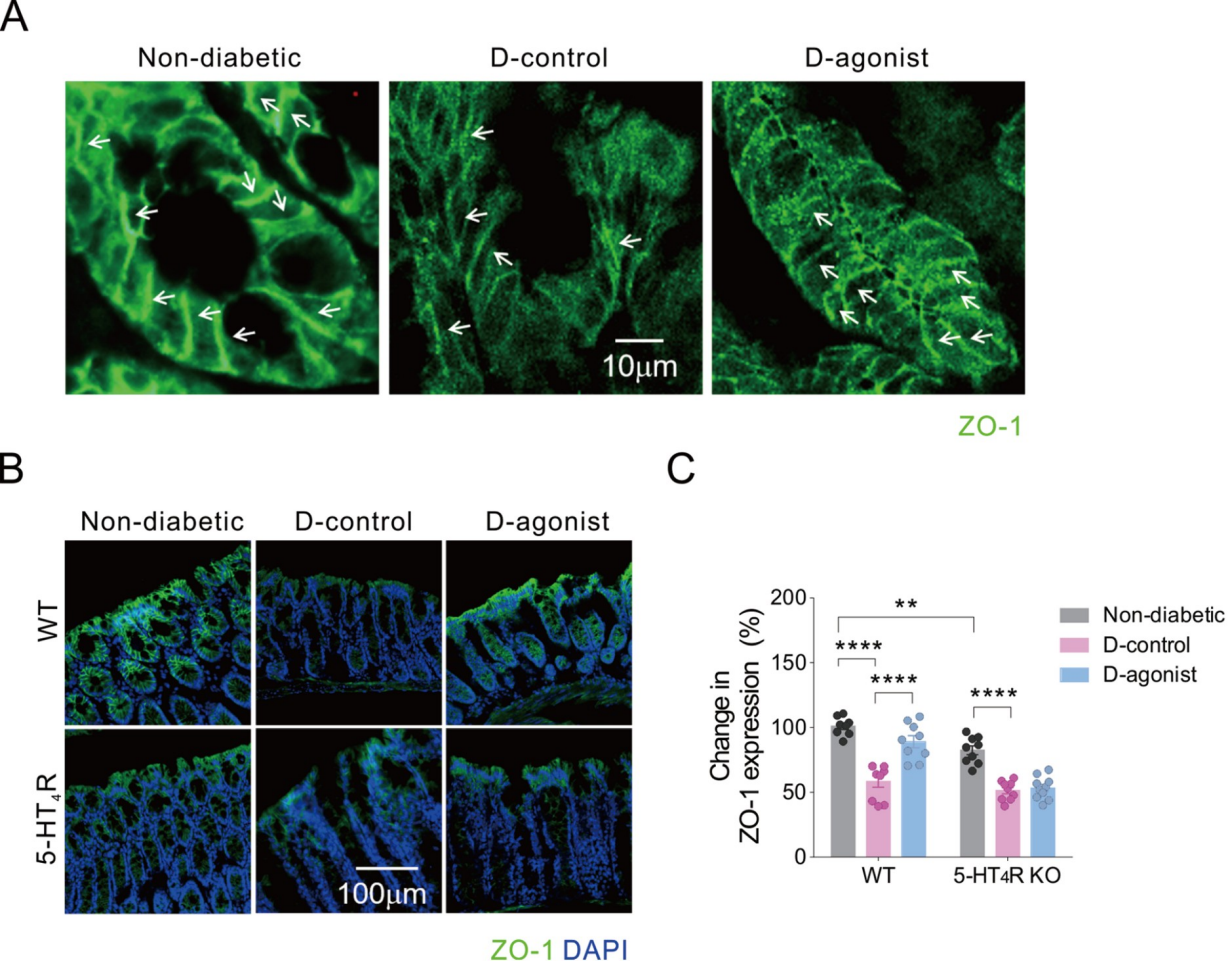
Figure 4 Activation of 5-HT _4_R impedes diabetes-induced decrease in immunofluorescence intensity of ZO-1 in the colon of mice (A) Representative photomicrographs of IF staining of ZO-1 in colon epithelial cells from WT mice, indicated by white arrows. Scale bar: 10 μm for all three images. (B) Representative photomicrographs of IF staining of ZO-1 (green) with DAPI (blue) in colon sections of WT and 5-HT4R KO mice. Scale bar: 100 μm for all six images. (C) Quantification of the IF intensity of ZO-1. **P<0.01, ****P<0.0001, two-way ANOVA with Tukey’s multiple comparison test (n=7–10 sections from 3–4 mice for each group). D: diabetic.

Thick line-like ZO-1 staining was regularly distributed throughout the lateral membrane of epithelial cells in the colon of diabetes-free mice. Thin line-like ZO-1 staining was irregularly localized on the lateral membrane of epithelial cells in the colon of mice with diabetes. Compared to diabetic control mice, a greater amount of thin-line ZO-1 staining was regularly distributed in colon epithelial cells in diabetic mice treated with the 5-HT _4_R agonist ( Figure 4A). Quantitative changes in IF staining of ZO-1 were similar to those of occludin and claudin-1 in both WT and 5-HT _4_R KO mice ( Figure 4B,C).

**Figure 5:**
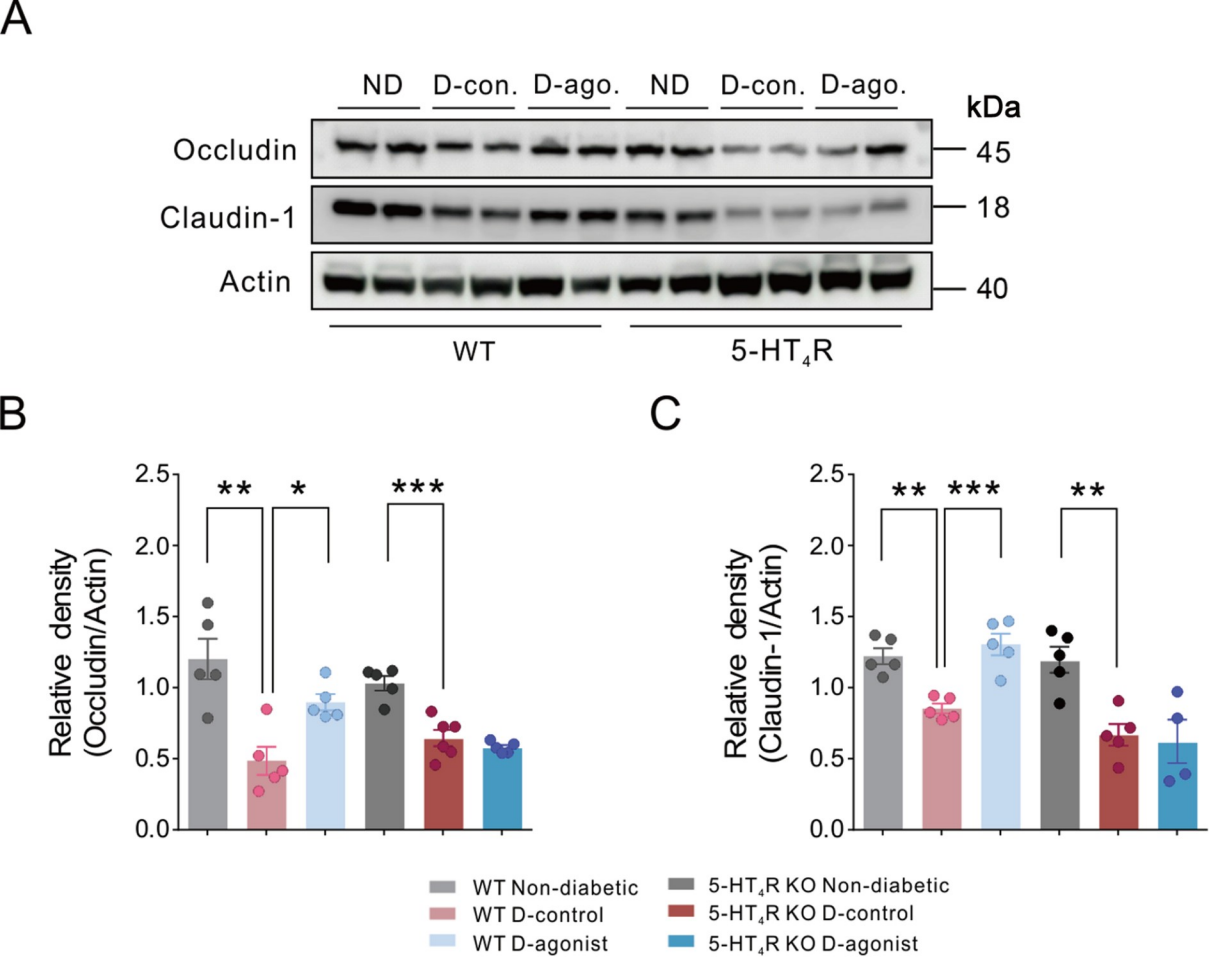
Figure 5 Activation of 5-HT _4_R limits diabetes-induced decrease in protein levels of occludin and claudin-1 in colonic mucosa in mice (A) Representative occludin and claudin-1 immunoblots in colonic mucosa from WT and 5-HT4R KO mice. ND: nondiabetic; D-con.: diabetic-control; D-ago.: diabetic-agonist. (B,C) Quantification of occludin and claudin-1 immunoblots. *P<0.05, **P<0.01, ***P<0.001, one-way ANOVA with Tukey’s multiple comparisons test (n=4‒6 mice for each group).

Western blot analysis showed that occludin and claudin-1 protein levels in colonic mucosa were lower in diabetic mice than in nondiabetic mice with or without 5-HT _4_R. As expected, stimulation of 5-HT _4_R by administration of its agonist enhanced occludin and claudin-1 protein levels in colonic mucosa in WT mice with diabetes, but the agonist did not block diabetes-induced decreases in occludin and claudin-1 protein levels in colonic mucosa in 5-HT _4_R KO mice ( Figure 5 ).

### 5-HT _4_R activation inhibits diabetes-triggered upregulation of MLCK, ROCK1 and pMLC in the colon

**Figure 6:**
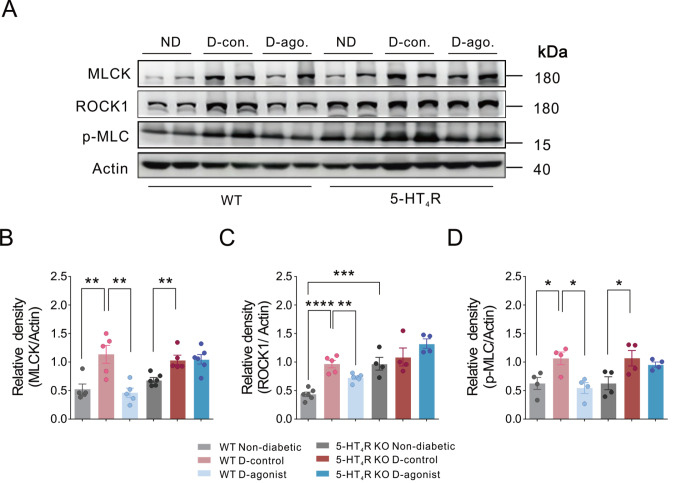
Figure 6 Activation of 5-HT _4_R suppresses diabetes-driven increase in the protein levels of MLCK, ROCK1 and p-MLC in the colonic mucosa of mice (A) Representative immunoblots of MLCK, ROCK1 and p-MLC in colonic mucosa from WT and 5-HT4R KO mice. ND: nondiabetic; D-con.: diabetic-control; D-ago.: diabetic-agonist. (B‒D) Quantification of MLCK, ROCK1 and p-MLC immunoblots. *P<0.05, **P<0.01, ***P<0.001, ****P<0.0001, one-way ANOVA with Tukey’s multiple comparisons test (n=4‒6 mice for each group).

To determine whether the 5-HT _4_R-mediated restoration of TJ proteins in the colonic mucosa in diabetes is related to the recovery of TJ integrity, we performed western blot analysis to examine the protein levels of MLCK, ROCK1, and p-MLC in the colonic mucosa after chronic treatment with the 5-HT _4_R agonist RS67333. In WT mice, the protein levels of MLCK, ROCK1, and p-MLC in the colonic mucosa were increased by STZ but not by vehicle. Chronic treatment with RS67333 dramatically inhibited diabetes-induced upregulation of MLCK, ROCK1, and pMLC in colonic mucosa compared to the control. To confirm that such effects induced by RS67333 occur via 5-HT _4_R, protein levels in mice lacking 5-HT _4_R were further tested. STZ still led to increases in the protein levels of MLCK, ROCK1, and p-MLC in the colonic mucosa compared to vehicle treatment, but RS67333 did not block the diabetes-induced increases in the protein levels of MLCK, ROCK1, or p-MLC in the colonic mucosa of 5-HT _4_R KO mice ( Figure 6).

## Discussion

Intestinal homeostasis is required for the physiological function of the intestinal epithelial barrier [ 17, 18]. Intestinal barrier dysfunction has been associated with a broad range of diseases, including inflammatory bowel disease [19], celiac disease [20] and nonalcoholic fatty liver disease [21], and is also correlated with systemic disorders of the outside intestine, such as diabetes [2], cancer [22] and neurodegeneration [23]. T1D induces intestinal barrier dysfunction, developing both commensal bacterial penetration [10] and pathogen infection [2]. In the current study, increased serum FITC-dextran and decreased TER, considered paracellular permeability dysfunction, were observed in STZ-induced diabetes. Our findings are consistent with the above studies where T1D develops intestinal barrier disruption.

The intestinal epithelium plays a complex role as a semipermeable barrier that supports the absorption of nutrients while preventing the translocation of harmful luminal contents into the body [17]. An interplay between structural components and molecular interactions in the intestinal epithelium maintains intestinal integrity to achieve such a “contradictory” role. The epithelial barrier consists of two major selective permeability pathways, one of which is the paracellular pathway that is regulated primarily by TJs and adherens junctions. TJs consist of multiple protein complexes of transmembrane, cytoplasmic plaque, cytoskeletal, and signaling proteins [6]. Occludin is the first identified transmembrane TJ protein [24]. Defects in barrier function are absent in mice lacking occludin [25], while enhanced TJ permeability occurs in epithelial monolayers with *occludin* knockdown [26]. The current study showed that occludin expression in the colon detected by both immunofluorescence staining and western blot analysis was decreased by STZ, confirming that T1D leads to occludin downregulation in the colon. Claudins, also transmembrane proteins, were initially identified in chicken liver junctional fractions [27]. Water and macromolecules will be lost when claudin-1, considered “sealing claudins”, is deleted [28]. Claudin-1 expression in the colon assessed by either immunofluorescence staining or western blot analysis was shown to be decreased by STZ in our study, indicating that T1D also results in the downregulation of claudin-1 in the colon. ZOs, multidomain scaffolding proteins, form a complex linking transmembrane and cytoskeletal proteins [29]. A previous study indicated that ZO-1, rather than ZO-2 or -3, plays a more important role in controlling TJ assembly [30]. Although the protein levels of ZO-1 could not be detected by western blot analysis due to the limitations of the antibody utilized in the current study, immunofluorescence staining results demonstrated that ZO-1 expression in the colon was reduced by STZ, suggesting that T1D also induces ZO-1 downregulation in the colon. Taken together, increased epithelial permeability and downregulation of occludin, claudin-1 and ZO-1 were observed in diabetic mice, indicating that T1D induces TJ barrier disruption.

The consensus is that 5-HT _4_R, one of the 5-HT receptors, is widely distributed in the gastrointestinal (GI) tract, including enteric neurons [31] and epithelial cells [ 8– 10]. 5-HT _4_R agonists have generally been used to treat constipation due to their prokinetic actions [32]. In particular, a 5-HT _4_R agonist has been used to treat gastroparesis in a murine model of diabetes [33] but also in patients with diabetic gastroparesis [34]. Recent studies highlight the protective actions of 5-HT _4_Rs expressed in epithelial cells. Epithelial 5-HT _4_R-mediated enhancement of wound healing processes is related to increased cell proliferation/migration and decreased cell apoptosis [ 9, 35]. Although it has been reported that diabetes results in a decline in the endogenous ligand 5-HT [36], STZ cannot influence 5-HT _4_R expression in colon epithelial cells [10]. In addition, protection against the penetration of commensal bacteria into the mucosa against diabetes occurs by promoting mucin 2 production when 5-HT _4_R is stimulated [10]. Here, we reported that activation of 5-HT _4_R stimulated by RS67333 alleviated increased epithelial permeability, characterized by higher serum FITC-dextran and lower TER in diabetic mice compared to nondiabetic mice. In addition, RS67333 may inhibit T1D-driven downregulation of TJ proteins, including occludin, claudin-1, and ZO-1. However, these effects induced by RS67333 failed to be achieved in mice lacking 5-HT _4_R. The protective effects of 5-HT _4_R activation were independent of glucose control because RS67333 was not capable of altering diabetes-induced changes in body weight and blood glucose. These results suggest that 5-HT _4_R activation can ameliorate T1D-induced TJ barrier disruption, expanding the above-mentioned knowledge about the protective actions of epithelial 5-HT _4_ R.

5-HT _4_R is a Gs protein-coupled receptor that specifically activates adenylyl cyclase to induce intracellular cAMP formation and subsequent activation of protein kinase A (PKA) [37]. PKA has been linked to downregulation of MLC phosphorylation through inhibition of MLCK and ROCK signaling in endothelial barrier function [ 13, 14]. In the current study, the 5-HT _4_R agonist RS67333 inhibited diabetes-induced upregulation of MLCK, ROCK1, and p-MLC in the colon, but the agonist failed to produce such an effect when *5-HT _4_ R* was knocked out, indicating that 5-HT _4_R-induced activation of PKA may lead to inhibition of MLCK/ROCK1 expression and downstream phosphorylation of MLC.

It is well known that phosphorylation of MLC leads to perijunctional actomyosin ring contraction, disrupting TJ protein assembly and TJ barrier integrity [4]. MLCK is a Ca ^2+^-calmodulin-activated serine/threonine kinase that phosphorylates MLC to promote actomyosin ring contraction [6]. MLCK1 is preferentially localized within the perijunctional actomyosin ring [38]. It has been reported that *MLCK1* knockdown decreases TJ permeability to regulate barrier function [39]. MLC phosphorylation can also be directly impacted by ROCKs, which are downstream effectors of GTP-binding Rho proteins and regulate perijunctional actomyosin ring dynamics [ 40, 41]. ROCK1 is widely distributed in the GI tract, liver, and lung [42]. Proinflammatory cytokines cause dysfunction of the intestinal TJ barrier via either MLCK1 or ROCK1, leading to increased intestinal permeability [ 43, 44]. The current study showed that T1D led to the downregulation of TJ proteins and upregulation of MLCK, ROCK1, and p-MLC, suggesting that disrupted TJ assembly induced by MLCK/ROCK1-p-MLC signaling pathways may contribute to the downregulation of TJ proteins in the colon in diabetes. However, the limitation of the current study is that we did not provide direct evidence that 5-HT _4_R activation protects the TJ barrier against diabetes by limiting the MLCK/ROCK1-pMLC signaling pathways.

Emerging evidence indicates potential therapeutic targeting of MLCK- and ROCK-mediated pathways [ 45, 46], but inhibitors targeting either MLCK or ROCK have some limitations. For example, systemic toxicity is associated with smooth muscle MLCK inhibition and lower selectivity. Consequently, therapeutic approaches to inhibit epithelial MLCK or ROCK are not currently clinically available. However, 5-HT _4_R agonists have been used in clinics. Intriguingly, our current study demonstrated that 5-HT _4_R activation not only inhibits the diabetes-induced upregulation of MLCK and ROCK1 but also restores the downregulation of TJ proteins in the colon in diabetes. These data suggest that 5-HT _4_R may become a potential therapeutic target to regulate MLCK or ROCK for the treatment of GI disorders related to intestinal barrier in clinic.

**Figure 7:**
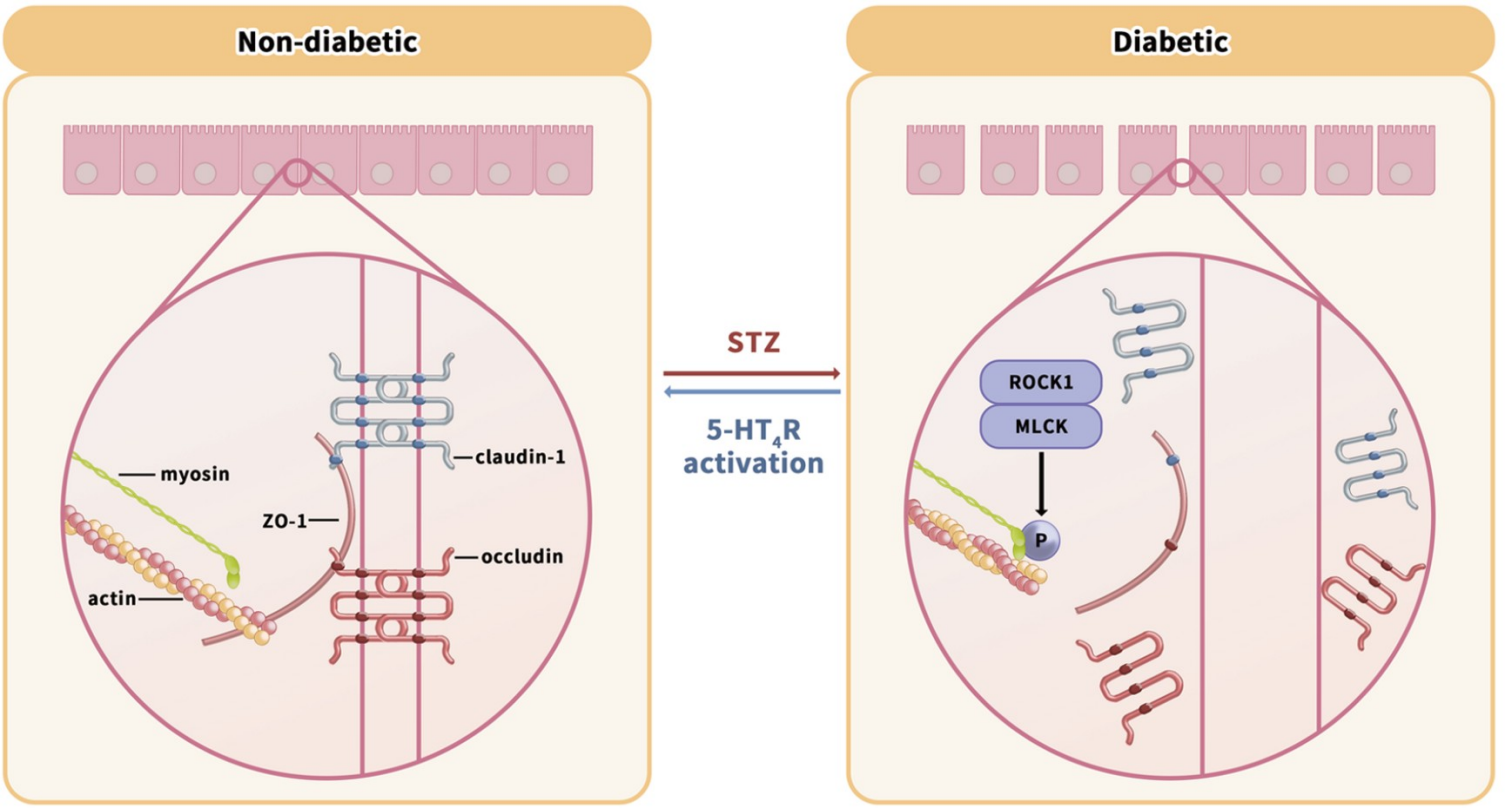
Figure 7 Schematic diagram of the role of 5-HT _4_R activation in the alleviation of diabetes-induced dysfunction of TJ barrier The potential protection of 5-HT4R activation in the TJ barrier from diabetes is mediated by inhibition of diabetes-induced upregulation of MLCK/ROCK1-p-MLC pathways, which promotes the contraction of the actomyosin ring, leading to disruption of TJ integrity and downregulation of TJ proteins.

In summary, this study demonstrated that a 5-HT _4_R agonist alleviated T1D-induced disruption of the TJ barrier, decreased the expressions of TJ proteins, including occludin, claudin-1, and ZO-1, and increased the expressions of MLCK, ROCK1, and p-MLC. However, such effects induced by the 5-HT _4_R agonist fail to occur in diabetic mice with 5-HT _4_R deletion. Our results suggest that 5-HT _4_R activation protects the TJ barrier against diabetes, possibly by limiting the MLCK/ROCK1-pMLC signaling pathways ( Figure 7).

## Supporting information

23212Figure_S1

## Supplementary Data

Supplementary data is available at *Acta Biochimica et Biophysica Sinica* online.
