# Supplementary figures and images for: Activation of the 5-hydroxytryptamine 4 receptor ameliorates tight junction barrier dysfunction in the colon of type 1 diabetic mice: Role of 5-HT 4R in the tight junction barrier of T1D
Source: Acta Biochim Biophys Sin (Shanghai). 2023 Sep 28;55(12):1874–83. doi: 10.3724/abbs.2023137 (PMC10753360; doi:10.3724/abbs.2023137)

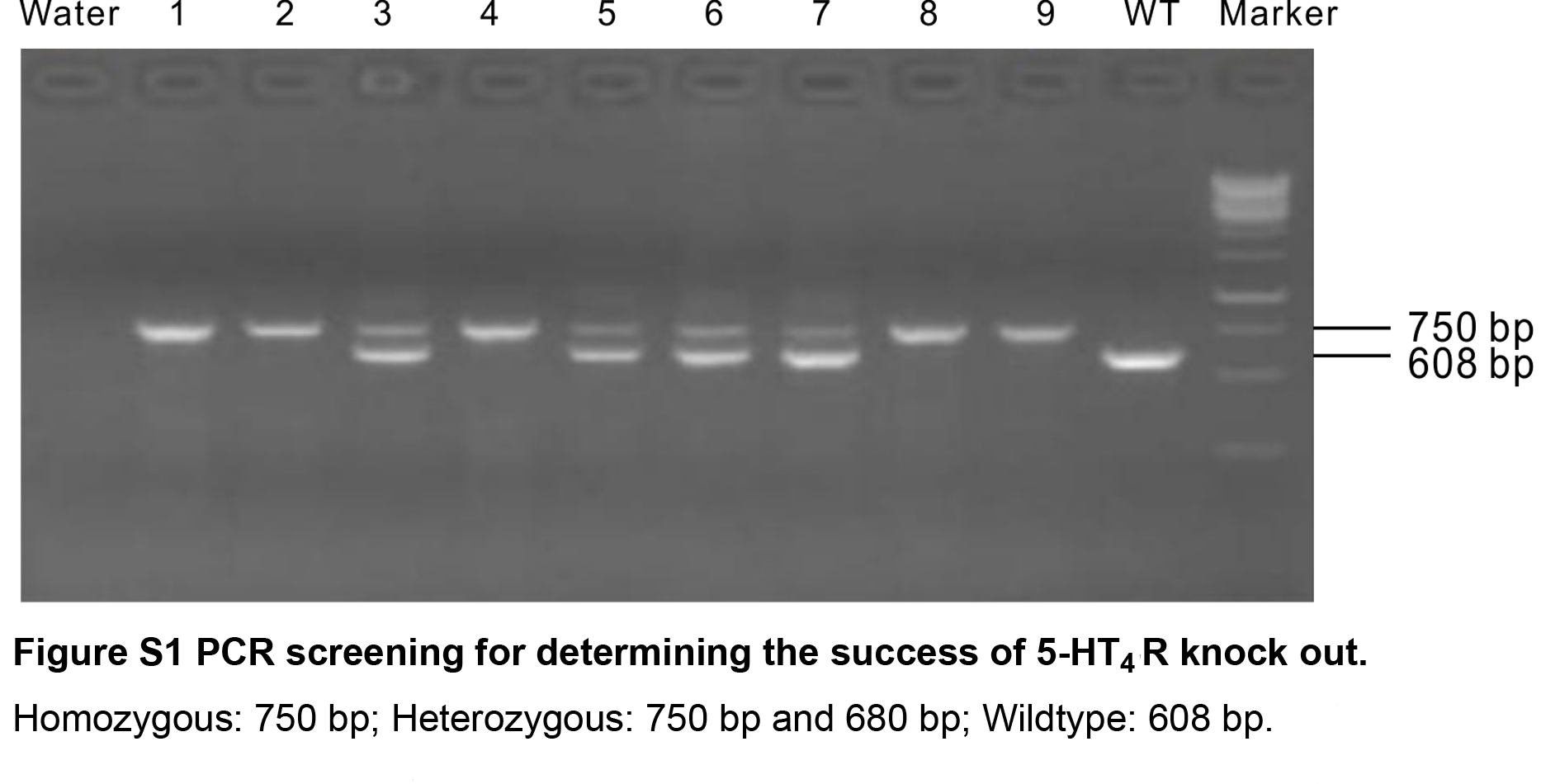

Supplement: 23212Figure_S1 [file 23212Figure_S1.tif]
